# The Minimum Methionine Requirement for Adults Aged ≥60 Years Is the Same in Males and Females

**DOI:** 10.3390/nu15194112

**Published:** 2023-09-23

**Authors:** Alyssa Paoletti, Paul B. Pencharz, Ronald O. Ball, Dehan Kong, Libai Xu, Rajavel Elango, Glenda Courtney-Martin

**Affiliations:** 1 Research Institute, Hospital for Sick Children, Toronto, ON M5G 1X8, Canada; alyssa.paoletti@sickkids.ca (A.P.); paul.pencharz@sickkids.ca (P.B.P.); 2 Department of Nutritional Sciences, University of Toronto, Toronto, ON M5S 1A8, Canada; 3 Department of Pediatrics, University of Toronto, Toronto, ON M5S 1X8, Canada; 4 Department of Agriculture, Food and Nutritional Science, University of Alberta, Edmonton, AB T6G 2P5, Canada; ball@ualberta.ca; 5 Department of Statistical Sciences, University of Toronto, Toronto, ON M5S 1X6, Canada; dehan.kong@utoronto.ca; 6 School of Mathematical Sciences, Soochow University, Suzhou 215006, China; lbxu@suda.edu.cn; 7 Department of Pediatrics, School of Population and Public Health, University of British Columbia, Vancouver, BC V6H 0B3, Canada; relango@bcchr.ubc.ca; 8 British Columbia Children’s Hospital Research Institute, British Columbia Children’s Hospital, Vancouver, BC V6H 3N1, Canada; 9 Faculty of Kinesiology and Physical Education, University of Toronto, Toronto, ON M5S 3J7, Canada

**Keywords:** indispensable amino acid, requirement, older adults, indicator amino acid oxidation, stable isotope

## Abstract

The minimum methionine requirement in the presence of excess dietary cysteine has not been determined in older adults. This study aimed to determine the minimum methionine requirement in healthy older adults using the indicator amino acid oxidation (IAAO) method. Fifteen healthy adults ≥ 60 years of age received seven methionine intakes (0 to 20 mg/kg/d) plus excess dietary cysteine (40 mg/kg/d). Oxidation of the indicator, L-[1-^13^C]phenylalanine (F^13^CO_2_), was used to estimate the mean minimum methionine requirement using a change-point mixed-effect model. There was no statistical difference between male and female requirement estimates, so the data were pooled to generate a mean of 5.1 mg/kg/d (R_m_^2^ = 0.46, R_c_^2^ = 0.77; *p* < 0.01; 95% CI: 3.67, 6.53 mg/kg/d). This is the first study to estimate the minimum methionine requirement in healthy older adults, which is the same between the sexes and as our lab’s previous estimate in young adults. The findings are relevant considering current recommendations for increased consumption of plant foods, which will help to establish the appropriate balance of methionine and cysteine intake required to satisfy the sulphur amino acid requirements of older adults.

## 1. Introduction

The current recommendation for sulphur amino acids (SAAs) in healthy adults of all ages is 15 mg/kg/d [1]. Recently, our group determined the total SAA requirement provided as dietary methionine (no dietary cysteine) in healthy adults ≥ 60 years of age to be 26.2 and 17.1 mg/kg/d for males and females, respectively [2]. These estimates have important implications considering the shift in nutrition recommendations for increased consumption of plant proteins [3,4]. Plant proteins are limiting in indispensable amino acids (IAAs), including methionine. In addition, animal data suggest that relative to animal foods, IAAs are less digestible from plant foods, particularly in older adults [5]. Plant-based diets would require older adults to eat a larger quantity of food to meet their IAA and protein needs, which increases the risk of obesity since energy needs decrease with age [6]. Therefore, meeting the SAA needs of older adults, especially in males, on a predominately plant-based diet must be carefully planned, particularly if a need for methionine predominantly drives the requirement for total SAAs. Work in animals [7,8,9] and young adults [10,11,12] demonstrated the ability of dietary cysteine to reduce the methionine requirement (i.e., cysteine sparing effect). This ability has also been demonstrated in young males using the indicator amino acid oxidation (IAAO) method [13,14].

There are only a handful of studies that have explored SAA metabolism in older adults [15,16]. In one study, Fukagawa and colleagues evaluated SAA kinetics at various intakes of methionine and cysteine in older adults [15] and showed that cysteine could only modestly reduce the methionine requirement. However, according to their study design, SAA intakes were based on the 1985 Food and Agriculture Organization’s (FAO) total SAA requirement of 13 mg/kg/d [17]. According to our recent total SAA requirement estimate of older adults [2], the intakes of SAAs in the Fukagawa study were too low to demonstrate a sparing effect. To explore the sparing effect of cysteine on the methionine requirement, the total SAA requirements must first be determined. In addition, establishing the minimum methionine requirement by providing excess dietary cysteine is necessary to understand the appropriate balance of dietary SAA intake that can meet the SAA needs of older individuals. Since various plant proteins are sufficient in cysteine [18] but limiting in methionine, this will be important in understanding how to satisfy the SAA needs of the elderly on a plant-based diet. Therefore, the primary objective of the current study was to determine the minimum methionine requirement in the presence of excess dietary cysteine of healthy adults ≥ 60 years of age using the IAAO method and to compare the estimates between older males and females. Since we observed a sex-based difference in the total SAA requirement, this will help to clarify whether older males have an increased demand for cysteine availability for glutathione (GSH) synthesis (i.e., total SAA requirement) or for methionine for methylation reactions (i.e., minimum methionine requirement). Our secondary objective was to measure concentrations of plasma glucose, insulin, C-reactive protein (CRP), homocysteine and amino acids (AAs), erythrocyte glutathione (GSH) concentration, and urinary sulphate concentration to explore their effect on the derived requirement estimate.

## 2. Materials and Methods

### 2.1. Subjects

**Figure 1:**
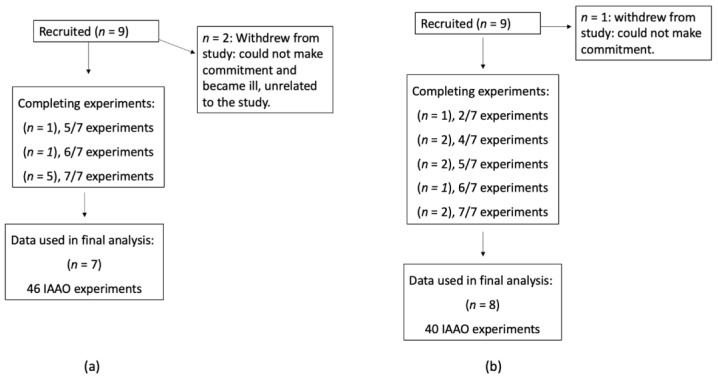
CONSORT flow diagram for older males (**a**) and females (**b**) ≥60 years old. Abbreviations: IAAO, indicator amino acid oxidation.

We screened and recruited a total of eighteen community-dwelling, healthy adults ≥ 60 years of age (9 males and 9 females). Before the study began, the study protocol was explained, and informed written consent was attained from each participant. Following recruitment, 2 male and 1 female subjects withdrew due to the time commitment and health issues unrelated to the study. A total of 15 older adults, 7 males and 8 females, completed the study; 7 subjects were studied at 7 intake levels of methionine, 2 subjects at 6 intake levels, 3 subjects at 5 intake levels, 2 subjects at 4 intake levels, and 1 subject at 2 intake levels for a total of 86 indicator amino acid oxidation (IAAO) experiments, completed in random order; details are presented in Figure 1.

The study took place between March 2022 and October 2022 at the Clinical Research Centre (CRC), The Hospital for Sick Children, Toronto, Canada. Exclusion criteria were chronic diseases and/or acute illness and/or use of medications that could affect protein and AA metabolism, abnormal ranges of fasting glucose, glycated hemoglobin A1c (HbA1c), urea, creatinine, weight loss or gain (>2.27 kg) in the past month, and study diet intolerance. Subjects with hypertension were not excluded if their blood pressure was regulated and their antihypertensive medications were consumed as prescribed by their physician. The Research Ethics Board at The Hospital for Sick Children approved all procedures. All subjects were provided financial compensation for their participation. This trial was registered at ClinicalTrials.gov (Identifier: NCT04595188).

### 2.2. Experimental Design

The study is a repeated measures design based on the minimally invasive IAAO protocol (21). Interested participants were screened for eligibility for a pre-study assessment on the phone. For those eligible, a fasted, pre-study assessment was conducted at SickKids. At the assessment, we collected subject’s weight, height, fat mass (FM), fat-free mass (FFM), resting energy expenditure (REE), and a questionnaire (i.e., medical history, supplement use, and physical activity levels). Continuous, open-circuit indirect calorimetry (QUARK RMR, COSMED USA Inc; Concord, CA, USA) was used to quantify REE. Skinfolds and BIA were used to measure FFM and FM, as previously described [19]. Waist and hip circumference measurements were taken and calculated as previously described [20]. A 10 mL blood sample was taken to measure fasting glucose, HbA1c, urea, and creatinine. These blood parameters were quantified to assess whether subjects had diabetes and to assess kidney function. Additionally, the blood sample was analyzed for vitamin B_6_, B_12_, folate, C-reactive protein (CRP), estradiol, testosterone, and GSH concentrations. Subjects were instructed to keep and bring with them to the assessment a completed 3 d (1 weekend and 2 weekdays) dietary food record to evaluate habitual dietary intakes. At the end of the pre-study assessment, subjects were fitted with an accelerometer (ActiGraph wGT3X-BT; Pensacola, FL, USA) to wear for 7 days to quantify their active energy expenditure. The accelerometry data and the calorimetry-derived REE were used to establish each participant’s individual total energy requirements.

In a repeated measures design, each level of methionine intake (0, 1, 3, 8, 14, 17, or 20 mg/kg/d) in the presence of excess dietary cysteine (40 mg/kg/d) was studied over a 3-day period (2 adaptation days followed by an IAAO study day). The levels of methionine intake were selected based on the previously derived total SAA requirement in the same age group. We provided excess cysteine at 10% above the requirement for total SAAs determined in older males [2]. To design the study to allow for a direct comparison of the breakpoints between sexes, the females also received the same amount of cysteine despite having a lower total SAA requirement. The intakes were carefully considered given our knowledge of The National Health and Nutrition Examination Survey (NHANES) analysis, which reported a positive association between the risk of cardiometabolic disease and quartile of SAA intake [21]. In that analysis, the highest quartile of SAA intake supplied a combined total of methionine and cysteine of 62.7 mg/kg/d. In this study, the maximum intake of methionine (20 mg/kg/d) and cysteine (40 mg/kg/d) equated to 60 mg/kg/d; however, while methionine and cysteine metabolism are intertwined, methionine is converted into homocysteine during transmethylation, whereas homocysteine is irreversibly condensed with serine to form cystathionine and cysteine. Therefore, the provision of dietary cysteine will contribute to the redistribution of homocysteine away from transulphuration and towards remethylation [8,14,22], which will contribute to a reduction in plasma homocysteine concentration and alleviate the risk of hyperhomocysteinemia. Moreover, while 30% of adults > 65 years have been reported to have elevated homocysteine levels, the majority of those incidences were attributed to deficiencies in vitamin B_12_, B_6_, and folate [23]. Therefore, we provided adequate vitamins/minerals based on the current DRI for the duration of the study.

Subjects were provided with a lactose-free milkshake maintenance diet (Scandishake; Scandipharm, Birmingham, AL, USA), which contained 1.0 g/kg/d and adequate energy (REE plus active energy expenditure measured by accelerometry) on the 2 days of adaptation [19,20]. After a 12 h fast (the IAAO study day), subjects consumed hourly meals that contained a randomly assigned test level of methionine and 40 mg/kg/d of cysteine. The 3-day experimental periods were separated by 1 to 2 weeks.

### 2.3. Study Diets

For the duration of the study, subjects received a daily 50+ multivitamin–mineral supplement (Centrum, Pfizer Consumer Healthcare, Mississauga, Canada) and 500 mg/kg/d choline supplement (Choline Bitartrate, Trophic, Richmondhill, Canada) to provide all micronutrient requirements based on current dietary reference intake (DRI) [1] and cofactors and interacting metabolites (i.e., folate, choline, vitamin B_6_, etc.) with the methionine cycle at adequate and constant intakes [24]. The adaptation diets were prepared as 4 equal meals/d. Subjects were not allowed to consume anything else except water and 1 cup of clear tea or coffee per adaptation day. Energy was provided as the sum of their REE plus their active energy expenditure measured by accelerometry.

On the third day, subjects came to the CRC following a 12 h overnight fast to perform the IAAO study day experiments. On each IAAO study day, diets were consumed as 8 hourly isocaloric meals, each meal representing one-twelfth of the daily requirement. The diet was supplied as a liquid formula composed of protein-free powder (PFD1; Mead Johnson, Evansville, IN, USA), orange-flavoured drink crystals (Fresh Plus Drink Crystals; WT Lynch Foods Limited, North York, Canada), grape seed oil, a crystalline AA mixture patterned after egg protein, and protein-free cookies [25]. The nitrogen content of the diets was adjusted according to the level of methionine intake with L-alanine to keep the diets isonitrogenous. Total protein was provided at 1.0 g/kg/d. Energy was provided as the sum of REE plus energy for sedentary activity [6]. Subjects were permitted water ad libitum on IAAO study days. Diets were designed to maintain weight and to provide macronutrient intakes within the acceptable macronutrient distribution range (AMDR).

For one randomly selected IAAO study day, subjects were instructed to arrive at the CRC to obtain a blood sample before consuming the test meals. A second blood sample was taken at the end of the IAAO study day. This was performed to assess the change in homocysteine concentration in response to varying methionine intakes in the presence of excess dietary cysteine.

### 2.4. Tracer Protocol

The minimally invasive oral tracer infusion protocol was used as previously described [26]. Beginning with meal 5, priming doses of 2.07 µmol/kg of NaH^13^CO_3_/kg and 3.99 µmol/kg L-[1-^13^C] phenylalanine were given with 7.99 µmol/kg/h of L-[1-^13^C] phenylalanine administered hourly until the eighth meal. The amount of phenylalanine provided as a tracer was subtracted from the dietary provision such that the total intake of phenylalanine was maintained at 25 mg/kg/d [27]. Tyrosine was provided at 160% of the estimated aromatic AA requirement (40 mg/kg/d) [28] to ensure that phenylalanine was not used to meet the demand for tyrosine [29]. Phenylalanine flux was not obtained. However, previous studies, including our most recent total SAA requirement determined in the same age group, showed that phenylalanine flux was unaffected by graded intakes of IAA and protein intake in older adults [2,20,27].

### 2.5. Sample Collection and Analysis

The pre-study assessment blood sample was sent to the clinical chemistry department at the Hospital for Sick Children for analysis of glucose, insulin, urea, creatinine, HbA1c, vitamin B_6_, vitamin B_12_, red blood cell folate, CBC for hematocrit to normalize erythrocyte GSH concentration, CRP, estradiol, and testosterone.

On each IAAO study day, breath and blood samples were collected. Before the tracer protocol began, five baseline breath samples were collected at 15 min intervals. Two and half hours after the start of the tracer protocol, when subjects reached isotopic steady state, plateau breath samples were collected. Breath sample collection and storage processes have been previously described [19]. Following meal 5, the volume of carbon dioxide production (VCO_2_) in mL/min was measured as previously described [20]. A blood sample was collected and sent to the clinical chemistry department at the Hospital for Sick Children at the end of each IAAO study day for measurement of glucose, insulin, CBC (for hematocrit to normalize erythrocyte GSH concentration), and CRP. An aliquot of the sample was also collected for measurement of erythrocyte GSH and plasma AA concentrations. In addition, at a randomly selected methionine intake level per subject, two blood samples were collected: one at the beginning and one at the end of the IAAO study day to measure changes in plasma homocysteine concentration in response to methionine intake. Urine samples were collected for measurement of urinary sulphate.

Serum glucose, urea, and creatinine concentrations were determined by a calorimetric assay as previously described [20]. Insulin was measured using an automated chemiluminescent micro-particle immunoassay (CMIA) on an automated analyzer (Abbott Architect i2000, Abbott Park, IN, USA) according to manufacturer instructions. HbA1c was measured using an enzymatic assay as previously described [20]. Vitamin B_12_ and red blood cell folate were quantified using a chemiluminescent microparticle intrinsic factor assay, a chemiluminescent microparticle folate binding protein assay, and a CMIA assay, respectively, on an automated analyzer (Abbott Architect Ci4100) according to manufacturer instructions. Vitamin B_6_, estradiol, and testosterone were analyzed at an external lab using LC-MS/MS [30] and CBC using the System XN 3000. The CRP Vario assay was used in the quantitation of CRP using Abbott Architect c Systems according to manufacturer instructions.

An aliquot of the IAAO study day blood sample was sent to The University of British Columbia for quantification of homocysteine, cysteine, and methionine concentrations using UPLC-MS/MS whereby the MS/MS (Waters Xevo TQS mass spectrometer; Waters Corporation, Milford, MA, USA) is coupled to UPLC (Waters H class UPLC; Waters Corporation, Milford, MA, USA). The LCMS was operated in MRM mode with ion transitions of 135.9–89.9 and 139.9–93.9 (d_4_-Hcy), 121.9–75.8 and 123.9–77.8 (d_2_ Cys), and 150.0–103.9 and 154.0–107.9 (d_4_ Met), for homocysteine, cysteine, and methionine, respectively. Briefly, 20 µL of plasma was transferred to a 1.5 mL Eppendorf tube containing 10 µL of internal standards (homocysteine-d_8_ (CDN Isotopes D-3030), cysteine-d_2_ (Cambridge Isotopes DLM-899), and methionine-d_4_ (CDN Isotopes D-3262), respectively, 10 µL of reducing solution (500mM dithiothreitol in 25 mM sodium hydroxide) was added, the mixture was vortexed, and samples were incubated at room temperature for 15 min to allow reduction of the disulphide bonds. Proteins were then precipitated by 100 µL acetonitrile (ACN) containing 0.2% (*v*:*v*) heptafluorobutyric acid (HFBA), and the samples were vortexed and centrifuged at 18,000× *g* for 10 min. One hundred microliters of supernatant was removed and diluted with 500 µL 0.2% (*v*:*v*) HFBA in water. Chromatographic separation of homocysteine, cysteine, and methionine was achieved using an Acquity UHPLC BEH C18, 2.1 × 50 mm column, 1.7 µm particle size with a guard column (Waters H class UPLC; Waters Corporation, Milford, MA, USA) with a mobile phase gradient of eluent A (deionized water with 0.2%HFBA and 0.1% formic acid) and B (methanol with 0.2%HFBA and 0.1% formic acid). All other plasma AA concentrations were quantified in our lab using UPLC (Acquity UPLC System; Waters Corporation, Milford, MA, USA). Briefly, 100 µL of methanol, 25 µL of sample, and 30 µL of internal standard (0.25 mM Norleucine) were added to a 1.5 mL Eppendorf tube and vortexed and centrifuged for 10 min. The supernatant was then transferred to derivatizing tubes, frozen at −80 °C for 30 min, and freeze-dried for 3 h. Next, samples were dried using a drying agent (30 µL of water, 10 µL of triethylamine; TEA, and 10 µL of methanol), vortexed, frozen at −80 °C for 15 min, and freeze-dried for 1 h. Samples were derivatized using a derivatizing agent (40 µL of water, 20 µL of TEA, 20 µL of PITC, and 140 µL of methanol), vortexed, frozen at −80 °C for 15 min, and freeze-dried for 1 hr. In the final step, 300 µL of diluent was added to samples, vortexed, and transferred to autosampler vials for chromatographic separation.

Aliquots of the pre-study assessment and IAAO study day blood samples were sent to the University of Toronto for erythrocyte GSH concentration analysis using an orbitrap mass spectrometer (Thermo Q-Exactive) in positive electrospray ionization mode coupled to a UHPLC (Thermo Scientific Ultimate 3000, Waltham, MA, USA). A urine sample was analyzed for sulphate by a spectrophotometric method reported by Swaroop [31] and modified according to Su and Gelius (2020) [32].

Continuous-flow isotope ratio mass spectrometer (CF-IRMS 20/20 isotope analyzer; PDZ Europa Ltd.; Northwich, UK) was used to measure expired ^13^CO_2_ enrichment, as previously described [19]. Enrichments were expressed as the APE compared with a reference standard of compressed CO_2_ gas.

### 2.6. Estimation of Isotope Kinetics

The rate of appearance of ^13^CO_2_ in breath following tracer oxidation (F^13^CO_2_, μmol/kg/h) was calculated according to Matthews et al. [33] using a factor of 0.82 to account for CO_2_ retained in the body’s bicarbonate pool [34]. At baseline and plateau, isotopic steady state in the tracer enrichment was represented by unchanging values of ^13^CO_2_ in breath.

### 2.7. Statistical Analysis

Since there is no formal sample size calculation available for breakpoint analysis, we select a sample size as previously described [2]. Using a power of 80% to achieve an *R*^2^ = 0.5 at a 5% significance level, a sample size of 5 males and 5 females is sufficient to estimate a breakpoint.

Statistical analyses were performed using R (R version *2023.06.0*+*421*) for Windows. Statistical analysis was performed on primary and derived variables, and data were expressed as means ± SEM. Significance was established at *p* < 0.05.

The minimum methionine requirement was estimated by applying a change-point mixed-effect regression model to the F^13^CO_2_ data, as previously described [2,20,35]. We applied the model to males and females separately. Since they were not statistically different, all data were combined to determine a breakpoint. The breakpoint was objectively determined by selecting the model that minimized the Akaike information criteria (AIC). To measure the goodness-of-fit of the model, we compute the *R*^2^ based on the Nakagawa and Schielzeth (2012) method [36]. The parametric bootstrap method by Staggs (2009), which assumes normality, was used to determine the variance around the breakpoint estimate and calculate a 95% CI: BP ± 1.96 × SE [37]. To assess whether the breakpoints were different between males and females in this study or between older adults in this study and young adults in the study of DiBuono et al. [38], the overlap in the CI was calculated as previously described [2,20].

For secondary outcomes, we consider a joint linear mixed effect model by treating each dependent variable (i.e., insulin, glucose, CRP, homocysteine, GSH, plasma AA, and urinary sulphate) as a response and methionine intake and sex as covariates. To account for the repeated measures design, we treated each subject as a random effect. Multiple comparisons were tested using Tukey’s post hoc test. Where there was an effect of sex, the difference in dependent variables between males and females at each intake level was tested using a two-sample *t*-test. 

A *t*-test was used to assess differences between male and female subject characteristics and dietary intakes. The minimum methionine requirement per kg FFM was derived from the mean minimum methionine requirement per kg body weight divided by the FFM, and differences between males and females were assessed using a *t*-test.

## 3. Results

### 3.1. Subject Characteristics

Fifteen healthy older males (aged 68.3 ± 2.09 years) and females (aged 70.6 ± 2.05 years) completed the study (Table 1). Body weight, height, FFM, WHR and REE, blood urea, creatinine, estradiol, and testosterone were significantly different between the sexes (*p* < 0.05). All subjects were not diabetic and had normal kidney function (Table 1). The 3-day dietary food records were completed by males (*n* = 6) and females (*n* = 7) (Table 2). Males had significantly higher protein intakes expressed in g compared to females (*p* < 0.05). However, after adjusting protein intake per kg of body weight, males and females had similar intakes and exceeded the RDA for protein of 0.8 g/kg/d [1]. Older female total and saturated fat intakes expressed as a % of kcal exceeded the upper end of the acceptable macronutrient distribution range (AMDR), whereas males’ total and saturated fat intakes fell within the AMDR. Males and females also exceeded the RDA for selenium, vitamin C, and B vitamins (B_6_ and B_12_). Interestingly, males had significantly higher selenium intakes compared to females (*p* < 0.05). Males met the RDA for vitamin E and folate; however, females did not. Nonetheless, the difference in intake between males and females was not statistically significant (*p* > 0.05). Additionally, both male and female habitual caloric intakes were consistent with their adaptation day calories. According to the current RDA for total SAAs of 19 mg/kg/d [1], the habitual intakes of total SAAs for males and females in this study met the current DRI.

On each IAAO study day, the average % calories from fat, carbohydrate, and protein were 34%, 50%, and 16%, respectively, for older males and females. The average energy intake on the study days was 2246 kcal/d for males and 1466 kcal/d for females.

### 3.2. L-[1-^13^C]Phenylalanine Oxidation

**Figure 2:**
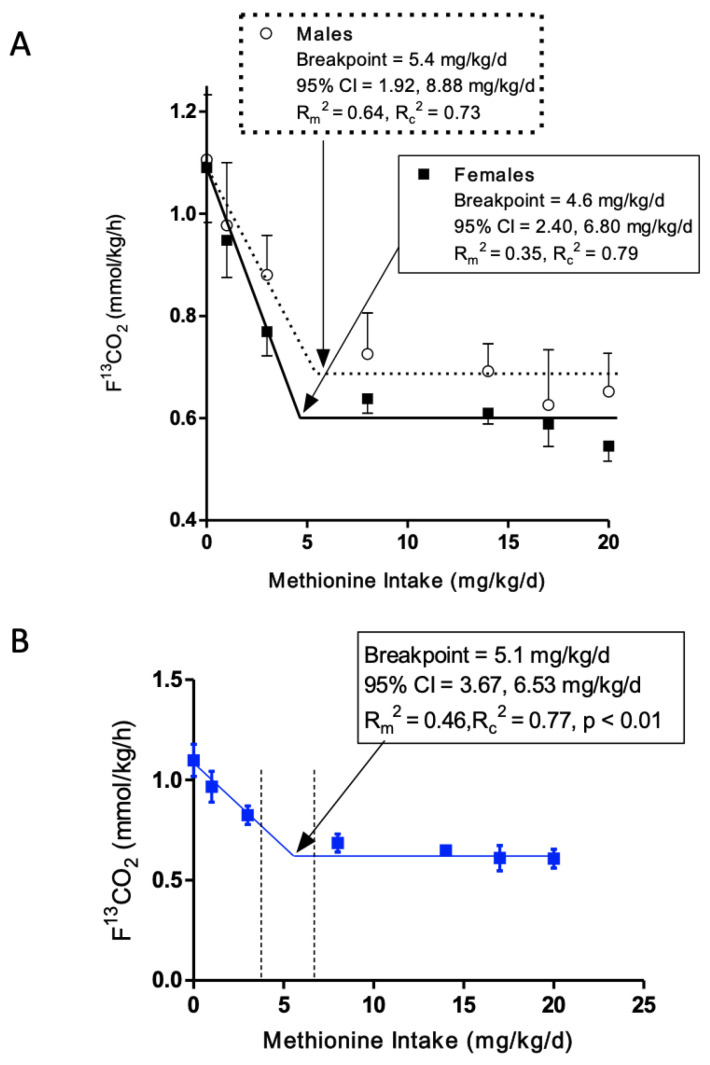
The effect of methionine intake with excess dietary cysteine on the production of ^13^CO_2_ from phenylalanine oxidation (F^13^CO_2_, mean ± SEM) by IAAO in older adults ≥ 60 years of age; *n* = 7 male and *n* = 8 female subjects; and 86 IAAO studies. Biphasic linear regression analysis of the F^13^CO_2_ data identified a breakpoint of 5.4 mg/kg/d for males (95% CI: 1.92, 8.88) and 4.6 mg/kg/d for females (95% CI: 2.40, 6.80), which represents the estimated mean minimum methionine requirement (**A**). The breakpoint for males and females combined was 5.1 mg/kg/d (*R*_m_^2^ = 0.46, *R*_c_^2^ = 0.77; *p* < 0.01) (**B**). The 95% CI of the combined estimate was 3.67 and 6.53 mg/kg/d. IAAO, indicator amino acid oxidation.

﻿ The rate of release of ^13^CO_2_ from L-[1-^13^C]phenylalanine oxidation (F^13^CO_2_) gradually decreased as methionine intake increased from 0 to 3 mg/kg/d in the presence of excess cysteine for both males and females. The remaining methionine intakes, 8 to 20 mg/kg/d, did not result in changes in F^13^CO_2_. Biphasic linear regression analysis of the F^13^CO_2_ data resulted in the identification of a breakpoint for the mean minimum methionine requirement of 4.6 mg/kg/d (*R*_m_^2^ = 0.35, *R*_c_^2^ = 0.79; *p* < 0.01) for females and 5.4 mg/kg/d (*R*_m_^2^ = 0.64, *R*_c_^2^ = 0.73; *p* < 0.01) for males (Figure 2A). The safe population level was estimated by determining the upper 95% CI of the breakpoints: 95% CI for females of 2.40 and 6.80, and 95% CI for males of 1.92 and 8.88 mg/kg/d.

Estimation of the overlap in the 95% CI of the breakpoints did reveal an interval of zero, meaning the null hypothesis of no difference was accepted. Therefore, we combined the data for both males and females and reanalyzed the combined data to estimate a breakpoint and 95% CI for the group. Biphasic linear regression analysis of the combined F^13^CO_2_ data resulted in a breakpoint of 5.1 mg/kg/d (*R*_m_^2^ = 0.46, *R*_c_^2^ = 0.77; *p* < 0.01, Figure 2B) and 95% CI of the estimate was 3.67 and 6.53 mg/kg/d.

### 3.3. Minimum Methionine Requirement Based on FFM

The minimum methionine requirement was 7.48 ± 0.28 and 7.26 ± 0.23 mg/kg FFM (mean ± SEM) for males and females, respectively (*p* > 0.05). The 95% CI of the estimates were 6.83 and 8.14 for males and 6.69 and 7.83 for females.

### 3.4. Comparison of Current Minimum Methionine Requirement Estimate of Older Adults to Young Adults

To compare older and young adult breakpoint estimates, we used our previously derived minimum methionine requirement in young adult males [13]. In that study, the mean minimum methionine requirement was found to be 4.6 mg/kg/d using breakpoint analysis in SAS, and the 95% CI was derived using Fillers Theorem. Since then, we have refined our statistical analysis using a method that takes into consideration the repeated measures design of the study and a parametric bootstrap method to calculate the 95% CI in R, which provides a narrower CI. Therefore, we reanalyzed the young adult male breakpoint using the new statistical approach in R, which provided a breakpoint of 3.5 mg/kg/d, and the 95% CI of the estimate was 1.01 and 5.99. This reanalyzed breakpoint and 95% CI of the minimum methionine requirement of young adult males was compared to the current breakpoint determined in older adults.

Estimation of the overlap in the 95% CI of the requirement estimate in the current study and the reanalyzed estimate of Di Buono et al. [13] did reveal an interval of zero, meaning the null hypothesis of no difference was accepted.

### 3.5. Effect of Methionine Intake and Sex on Secondary Outcomes

Concentrations of plasma glucose, insulin, CRP, erythrocyte GSH, and urinary sulphate (mean ± SEM) were unaffected by graded intakes of methionine (*p* = 0.3697, *p* = 0.9612, *p* = 0.4756, *p* = 0.6974, and *p* = 0.2812, respectively; Figure S1). There were no differences in glucose, CRP, GSH, and urinary sulphate concentrations between males and females (6.07 ± 0.02 and 5.92 ± 0.02 mmol/L, *p* = 0.5734; 1.79 ± 0.21 and 1.54 ± 0.04 mg/L, *p* = 0.9888; 2.71 ± 0.11 and 2.77 ± 0.08 mmol/L, *p* = 0.7813, and 39.7 ± 4.71 and 42.8 ± 4.02 µmol/µmol_creatinine_, *p* = 0.8288, respectively). However, males had significantly higher fed-state plasma insulin concentrations compared to females at methionine intakes of 3 (227 ± 17.9 vs. 116 ± 6.44 pmol/L, *p* = 0.0272), 8 (233 ± 15.8 vs. 96.8 ± 6.68 pmol/L, *p* = 0.0142), and 14 (227 ± 10.9 vs. 101 ± 2.48 pmol/L, *p* = 0.0004) (Figure S1).

**Figure 3:**
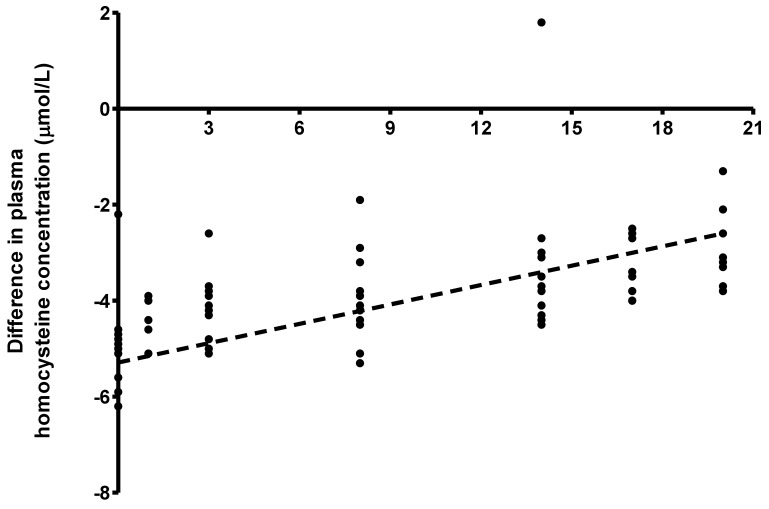
Difference between plasma homocysteine concentrations at baseline (fasted state) and in response to graded intakes of methionine (fed state) (*n* = 8 females studied for a total of 38 observations and *n* = 7 males studies for a total of 38 observations). A linear increase in the difference in plasma homocysteine concentration in response to graded intakes of methionine (*R_m_*^2^ = 0.34, *R*_c_^2^ = 0.38; *p* < 0.001) was determined using a joint linear mixed effect model. The regression equation for the calculation of the difference from baseline in plasma homocysteine concentration in response to methionine intake is given by y = −4.61 + 0.0927x (*p* < 0.001).

The difference between plasma homocysteine concentration (mean ± SEM) at baseline and in response to graded intakes of methionine was significantly affected by methionine intake (*p* < 0.001) but not sex (*p* = 0.777). Post hoc analysis revealed significant differences at methionine intakes 0 compared with 14 (−5.01 ± 0.26 vs. −3.25 ± 0.49 µmol/L, *p* < 0.001), 0 compared with 20 (−5.01 ± 0.26 vs. −2.90 ± 0.24 µmol/L, *p* < 0.001) and 3 compared with 20 (−4.23 ± 0.21 vs. −2.90 ± 0.24 µmol/L, *p* = 0.0249). The plasma homocysteine concentration increased linearly in response to graded intakes of methionine (y = −4.61 + 0.0927x, *R_m_*^2^ = 0.34, R_c_^2^ = 0.38; *p* < 0.001, Figure 3).

Plasma AA concentrations (mean ± SEM) were unaffected by sex (*p* > 0.05) and methionine intake (*p* > 0.05) except for methionine concentration, which was affected by methionine intake (*p* < 0.001) (Table S1).

## 4. Discussion

To our knowledge, this is the first study to determine the dietary minimum methionine requirement of healthy adults ≥ 60 years of age. We derived the EAR and RDA for minimum methionine of 5.4 and 8.8 mg/kg/d for males and 4.6 and 6.8 mg/kg/d for females. No sex differences in the requirement estimates were found on a body weight or FFM basis. As a result, we combined all data to derive a combined EAR of 5.1 and RDA of 6.53 mg/kg/d for minimum methionine in healthy adults ≥ 60 years of age.

The current estimates are analogous to our reanalyzed minimum methionine requirement of 3.5 mg/kg/d in healthy young adults using the IAAO method [13]. Thus, the physiological demand for methionine for protein synthesis, as a precursor for cysteine and as a donor of methyl groups for various methylation reactions, is the same in young and older adults [9]. This finding provides preliminary evidence to reject the hypothesis for increased dietary methionine intake to alleviate global hypomethylation observed in older adults [40]. Our group has recently determined the TSAA requirement as dietary methionine in the absence of dietary cysteine in a similar group of adults ≥ 60 years of age [2]. In that study, we derived a total SAA requirement of 26.2 mg/kg/d for males and 17.1 mg/kg/d for females. The total SAA requirement estimate for older males was significantly higher than for older females and young adults. Considering the current data, this suggests that an increased demand for dietary cysteine drives the increased total SAA requirement for older males. In the present study, we evaluated sex hormones at baseline to explore whether they may play a role in the observed sex effect on total SAA requirement. We found that concentrations of both testosterone and estradiol fell within normal ranges [41,42] for age, and neither sex hormone had a significant effect on F^13^CO_2_ response and likely did not play a role.

It has been well established that SAAs are essential for protein synthesis and the synthesis of the most abundant in vivo antioxidant, GSH [43,44]. To this end, cysteine is considered the rate-limiting substrate for GSH synthesis. It has been demonstrated that older adults have less capacity to make GSH, which was associated with higher plasma oxidative stress markers compared to young adults [45]. Upon supplementation with dietary cysteine, GSH synthesis was increased, and oxidative stress markers were reduced [45], suggesting that more SAA substrate is required for older adults to support GSH synthesis. Older males and females were not compared in that study; therefore, sex differences in GSH kinetics warrant further investigation. In the current study, we measured erythrocyte GSH concentration at each intake level and found no sex-based or intake effects. This finding can be attributed to the fact that its kinetics influences GSH concentrations, and thus, a static measure like concentration does not provide information regarding rates of GSH synthesis or breakdown [46]. Also, we were providing a constant and excess quantity of dietary cysteine, which was most likely not limiting at any intake for GSH synthesis. Therefore, the similar and unchanging GSH concentrations we observed are unsurprising from a physiological perspective. Nonetheless, physiological studies have shown that older male mice experience a larger decline in GSH concentration compared to females in various tissues [47,48]. Additionally, in older animal models, the rate-limiting enzyme for GSH synthesis called y-glutamylcysteine synthetase (y-GCS) has a weakened affinity for cysteine, suggesting that more cysteine is needed to synthesize GSH. Importantly, it has been shown that in older male mice, there is a more dramatic decline in hepatic y-GCS protein levels, corresponding to a greater decrease in GSH concentration compared to females [47,48,49]. These observations may partly explain the increased need for cysteine in older males compared to females, requiring greater substrate availability (cysteine) to make GSH.

In the current study, males had significantly higher WHR than females, which may be of clinical significance. WHR is a surrogate measure of central adiposity [50,51] and is positively associated with inflammation [52], oxidative stress [53], and insulin resistance [54,55,56]. We also found that males had significantly higher fed-state plasma insulin concentrations compared to females, which may signify greater insulin resistance in older males than females. Indeed, higher visceral adiposity in males has been associated with elevated postprandial insulin [57]. A report on the role of insulin in regulating GSH suggests that in insulin-resistant states, there is a reduction in the activity of y-GCS [58,59]. Since our older male participants appear to be more insulin resistant than the females, their y-GCS enzymatic activity may be more compromised, and thus they require more cysteine to make GSH. This line of reasoning is supported by data from older adult males showing that they generally have greater adiposity, insulin resistance, and oxidative stress [60] compared to females; while we did not measure oxidative stress in this study, there is evidence to support higher oxidative stress markers in older males compared to females [60,61]. Nonetheless, further investigation into the role of body composition, insulin resistance, and oxidative stress on the sex difference in total SAA requirement of older adults is warranted.

We provided excess cysteine at 10% above the requirement for total SAAs determined in older males, which permitted a breakpoint comparison between older males and females. Therefore, older females received a larger magnitude of excess dietary cysteine; however, plasma homocysteine concentrations did not differ between sexes and were within safe physiological ranges. Additionally, cysteine concentrations were comparable between sexes and intakes, demonstrating a tight regulation of plasma cysteine concentration in healthy adults > 60 years of age. These observations suggest that females incurred no harm despite consuming a larger excess of dietary cysteine and that older adults maintain tight regulation of SAA metabolism in the context of a healthy diet (i.e., adequate vitamins/minerals). An epidemiological study reported a positive association between SAA intake and cardiometabolic risk scores; however, the authors reported that the healthy eating index score was lowest in participants in the highest SAA quartile [21]. Upon further investigation, those in the highest SAA quartile consumed approximately double the amount of animal protein and/or meat (serving per day) compared to the other quartiles. Thus, while saturated fat intakes were not reported, the observed association may be confounded by a higher saturated fat intake manifested by the lower healthy eating index and not SAA intake per se.

The mean methionine requirement in the presence of dietary cysteine in the current study for males and females is 20% and 30%, respectively, of the mean total SAA requirement (diet devoid of cysteine) for older males and females found in our previous study [2]. This demonstrates an 80 and 70% sparing effect of cysteine on the methionine requirement in older males and females, respectively. The magnitude of cysteine sparing in the current study falls within the cysteine sparing range previously observed in humans [7,11,12,24]. This finding provides evidence that cysteine can fulfill part of the SAA requirement and thus reduce the amount of dietary methionine in younger as well as older adults. This is important to consider when devising nutrition recommendations, particularly in older adults who might be more susceptible to deficient intakes of SAA on a plant-based diet given their higher requirement for SAA, particularly in males.

The current findings have imperative implications considering the recent change in nutrition guidelines for increased plant protein consumption [3,4]. A strict plant-based diet may be more environmentally sustainable [4,62] and provide various health benefits [63,64]; however, it may not be suitable to meet the SAA requirements of older adults, specifically males, if not properly planned. However, various plant proteins are sufficient in cysteine [18] and, due to the cysteine-sparing effect observed in the current study, may be able to supply the increased demand for cysteine in older adult males. This emphasizes the need to reconsider the criteria for assessing the protein quality of foods in terms of their SAA content to understand more appropriately which combination of plant foods can supply the appropriate balance of SAAs, such as methionine and cysteine.

In summary, we have determined the minimum methionine requirement of older adults using the IAAO method. The EAR and RDA for minimum methionine were 5.1 and 6.53 mg/kg/d for older adults. The requirement for older adults is not different from the requirement estimates for young adults. However, future research is needed to investigate the sex difference in SAA requirements and the apparent increased demand for cysteine, particularly in older adult males. The results have important implications in light of the national and international transition to plant-based diets, which are limiting in SAAs. They will be necessary in devising dietary recommendations that will ensure older adults are meeting their specific needs for optimal health and well-being.

## Figures and Tables

**Table 1:** Subject characteristics of participants at baseline.

	Value ^1^	
Baseline Characteristics	Females (*n* = 8)	Males (*n* = 7)
Age (years)	70.6 ± 2.05	68.3 ± 2.09
Weight (kg)	60.6 ^a^ ± 3.33	85.5 ^b^ ± 5.74
Height (cm)	159 ^a^ ± 2.56	177 ^b^ ± 2.10
BMI (kg/m^2^)	23.9 ± 1.23	27.3 ± 1.67
FFM-SF ^2^ (kg)	41.8 ^a^ ± 2.22	58.0 ^b^ ± 2.64
FFM-BIA ^3^ (kg)	43.0 ± 1.67	62.1 ± 2.59
Fat -SF ^2^ (%)	29.3 ± 2.38	31.3 ± 2.73
Fat-BIA ^3^ (%)	28.9 ± 4.10	26.3 ± 3.00
WHR	0.85 ^a^ ± 0.03	0.97 ^b^ ± 0.04
REE ^4^ (kcal/d)	1229 ^a^ ± 48.1	1698 ^b^ ± 81.5
Blood Hb A1C (%)	5.44 ± 0.08	5.39 ± 0.12
Fasting Blood Glucose (mmol/L)	5.25 ± 0.19	5.37 ± 0.20
Fasting Insulin (pmol/L)	42.0 ± 8.55	45.6 ± 7.78
HOMA-IR ^5^	1.49 ± 0.29	1.63 ± 0.35
Fasting Blood Urea (mmol/L)	5.03 ^a^ ± 0.38	6.33 ^b^ ± 0.45
Fasting Blood Creatinine (µmol/L)	65.5 ^a^ ± 3.5	78.6 ^b^ ± 4.6
C-reactive Protein (mg/L)	1.43 ± 0.56	1.07 ± 0.15
Vitamin B12 (pmol/L)	477 ± 117	291 ± 49.7
Vitamin B6 (nmol/L)	155 ± 80.8	60.1 ± 25.6
Red Blood Cell Folate (nmol/L)	963 ± 80.2	885 ± 113
Testosterone (nmol/L)	1.07 ^a^ ± 0.21	20.0 ^b^ ± 3.18
Estradiol (pmol/L)	20.0 ^a^ ± 3.42	70.8 ^b^ ± 10.8
GSH (mmol/L)	2.79 ± 0.28	2.87 ± 0.36

^1^ All values are means ± SEM. Values with different superscripts were significantly different, with *p* < 0.05 determined by *t*-test. Abbreviations: BMI, body mass index; FFM, fat-free mass; HbA1c, glycated hemoglobin; REE, resting energy expenditure; SF, skinfold; and WHR, waist-to-hip ratio. ^2^ Determined by SF analysis. ^3^ Determined using BIA analysis. ^4^ Determined by open-circuit indirect calorimetry. ^5^ HOMA-IR: fasting insulin (mU/L) × fasting glucose (mmol/L)/22.5 [39].

**Table 2:** Habitual dietary intakes of participants.

	Value ^1^			
Nutrient	Females (*n* = 7)	% RDA	Males (*n* = 6)	% RDA
Calories (kcal/d)	1558 ± 149	-	2464 ± 135	-
Calories (kcal/kg/d)	25.6 ± 0.92	-	28.5 ± 1.56	-
Protein (g)	58.2 ± 4.53	-	111 ± 10.4	-
Protein (g/kg/d)	0.99 ± 0.10	124	1.37 ± 0.27	171
Total sulphur amino acid (mg/kg/d)	19.1 ± 2.43	100	28.6 ± 8.15	151
Total Fat ^2^ (g)	66.4 ± 4.85	110	90.2 ± 4.90	94
Saturated fat ^2^ (g)	19.5 ± 1.63	113	23.6 ± 3.24	86
MUFA (g)	26.0 ± 2.56	-	33.3 ± 3.22	-
PUFA (g)	14.7 ± 1.63	-	19.7 ± 2.40	-
Trans fat (g)	0.98 ± 0.41	-	0.63 ± 0.16	-
Selenium (µg)	77.1 ^a^ ± 18.3	140	146 ^b^ ± 16.3	266
Vitamin C (mg)	110 ± 13.2	147	185 ± 39.6	206
Vitamin E (mg)	9.07 ± 1.22	61	14.6 ± 2.81	97
Folate (µg)	324 ± 27.9	81	492 ± 89.5	123
Vitamin B_6_ (µg)	3.44 ± 2.10	229	2.81 ± 0.35	165
Vitamin B_12_ (µg)	3.35 ± 0.63	140	7.35 ± 1.90	306

^1^ All values are means ± SEM. Values with different superscripts were significantly different, with *p* < 0.05 determined by *t*-test. All intakes are based on food and beverage consumption and supplements. To calculate the %RDA, means of each nutrient consumed are compared to their respective RDA multiplied by 100. Abbreviations: MUFA, monounsaturated fatty acid; PUFA, polyunsaturated fatty acid; and RDA, recommended dietary allowance. ^2^ Total and saturated fat intakes in g were converted to kcal (9 kcal/g fat) to compare intakes to the acceptable macronutrient distribution range.

## Data Availability

The data presented in this study are available on request from the corresponding author. The data are not publicly available for privacy reasons.

## Supplementary Materials

The following supporting information can be downloaded at https://www.mdpi.com/article/10.3390/nu15194112/s1. Figure S1: Concentrations of glucose (a), insulin; with significant differences between males and females at protein intakes 0.8 (*p = 0.0496), 1.3 (^#^p = 0.0398) and 1.5 (†p = 0.0249). (b), CRP (c), intracellular GSH (d), and urinary sulphate excretion (e) in older adults who participated in the study to determine the minimum methionine requirement of adults ≥ 60 y in good health; Table S1: Plasma amino acid concentrations of healthy older adults who participated in the study to determine the minimum methionine requirement.
