# Supplementary material for: The Minimum Methionine Requirement for Adults Aged ≥60 Years Is the Same in Males and Females
Source: Nutrients. 2023 Sep 23;15(19):4112. doi: 10.3390/nu15194112 (PMC10574673; doi:10.3390/nu15194112)
Supplement: Supplementary file 1 [file nutrients-15-04112-s001.zip › Supplementary Figure S1.pptx]

## Slide 1
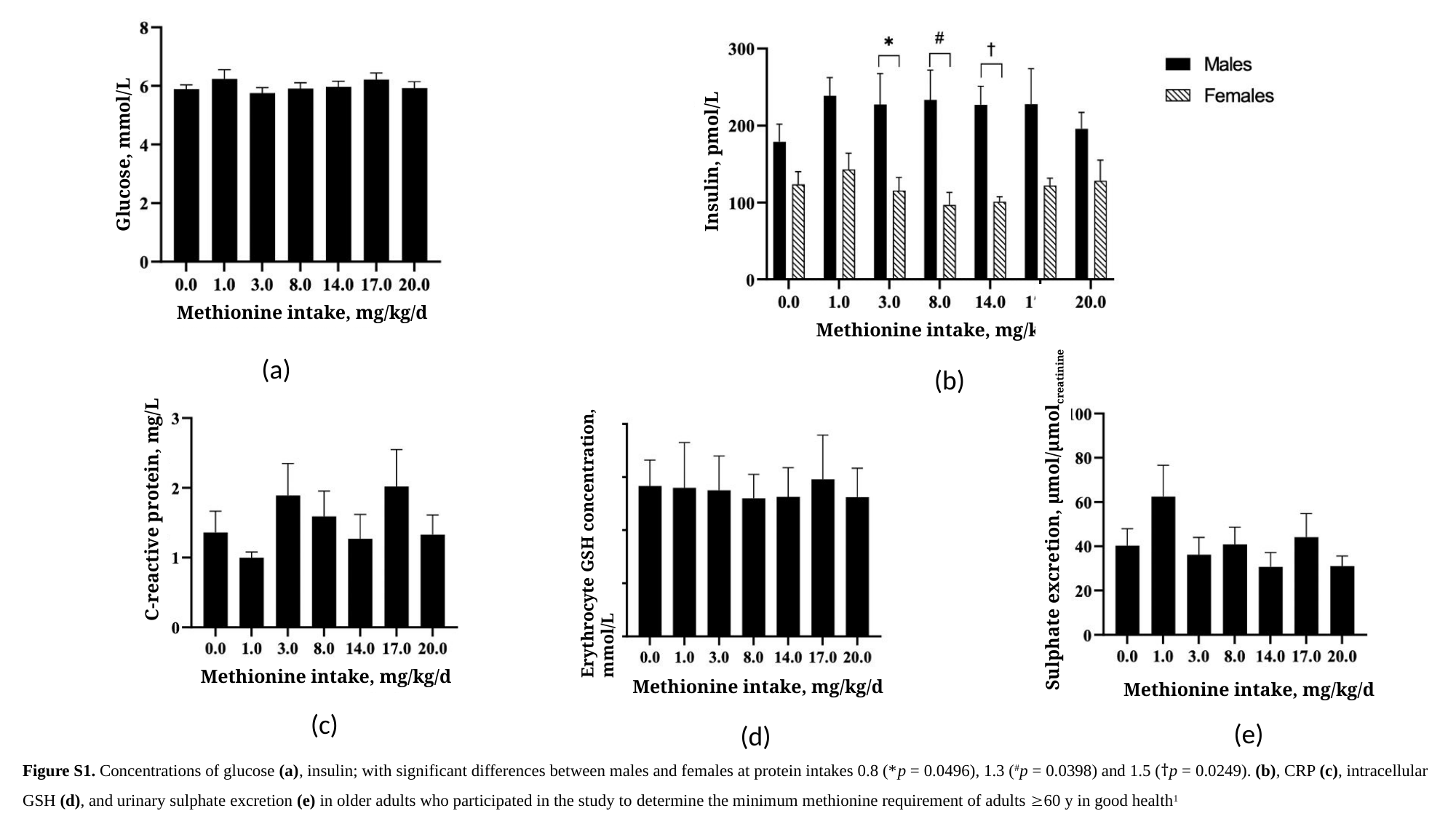

Glucose, mmol/L
Insulin, pmol/L
Methionine intake, mg/kg/d
Methionine intake, mg/kg/d
(a)
(b)
C-reactive protein, mg/L
Sulphate excretion, µmol/µmolcreatinine
Erythrocyte GSH concentration, mmol/L
Methionine intake, mg/kg/d
Methionine intake, mg/kg/d
Methionine intake, mg/kg/d
(c)
(e)
(d)
Figure S1. Concentrations of glucose (a), insulin; with significant differences between males and females at protein intakes 0.8 (*p = 0.0496), 1.3 (#p = 0.0398) and 1.5 (†p = 0.0249). (b), CRP (c), intracellular GSH (d), and urinary sulphate excretion (e) in older adults who participated in the study to determine the minimum methionine requirement of adults ≥60 y in good health1
