# Supplementary material for: The Minimum Methionine Requirement for Adults Aged ≥60 Years Is the Same in Males and Females
Source: Nutrients. 2023 Sep 23;15(19):4112. doi: 10.3390/nu15194112 (PMC10574673; doi:10.3390/nu15194112)
Supplement: Supplementary file 1 [file nutrients-15-04112-s001.zip › Supplementary Table S1.pdf]

Table S1. Plasma amino acid concentrations of older adults who participated in the study to determine the minimum methionine requirement of adults ≥60 y in good health[1]

**Amino Acids**[1], μmol/L	**Methionine Intake,** mg/kg/d						
	0	1	3	8	14	17	20
Alanine	622 ± 69	625 ± 167	778 ± 111	722 ± 149	676 ± 76	675 ± 93	698 ± 71
Arginine	144 ± 14	145 ± 37	170 ± 22	151 ± 29	154 ± 16	146 ± 24	153 ± 17
Cysteine[2]	309 ± 8.0	295 ± 6.8	308 ± 8.8	297 ± 10	314 ± 11	304 ± 11	302 ± 11
Citrulline	53 ± 7.4	51 ± 14	71 ± 10	65 ± 13	58 ± 8.3	53 ± 9.2	52 ± 8.1
Glutamate	93 ± 16	114 ± 42	105 ± 15	110 ± 21	99 ± 15	79 ± 11	84 ± 12
Glutamine	1609 ± 178	1563 ± 432	1988 ± 256	1830 ± 397	1680 ± 188	1686 ± 240	1672 ± 196
Glycine	296 ± 47	285 ± 64	405 ± 54	304 ± 66	377 ± 58	308 ± 45	341 ± 37
Histidine	114 ± 12	121 ± 30	138 ± 16	129 ± 25	122 ± 11	121 ± 14	132 ± 13
Isoleucine	200 ± 27	208 ± 64	214 ± 36	210 ± 51	190 ± 23	170 ± 29	177 ± 19
Leucine	254 ± 37	260 ± 84	267 ± 45	261 ± 67	216 ± 26	207 ± 37	198 ± 26
Lysine	266 ± 26	268 ± 69	321 ± 39	293 ± 57	271 ± 29	268 ± 36	299 ± 27
Methionine[2]	6.3 ± 1.1[a]	7.5 ± 1.4[a]	12 ± 2.1[a,b]	18 ± 1.8[b]	29 ± 4.1[c]	30 ± 2.2[c]	34 ± 1.6[c]
Phenylalanine	131 ± 14	121 ± 29	149 ± 16	133 ± 25	119 ± 10	122 ± 13	141 ± 9.5
Proline	294 ± 40	304 ± 81	358 ± 50	329 ± 64	308 ± 36	285 ± 43	309 ± 34
Serine	193 ± 23	183 ± 48	250 ± 36	194 ± 41	205 ± 26	189 ± 32	174 ± 18
Threonine	204 ± 24	194 ± 53	266 ± 39	223 ± 44	217 ± 26	210 ± 37	219 ± 28
Tyrosine	122 ± 14	122 ± 34	133 ± 16	135 ± 30	110 ± 11	109 ± 15	115 ± 12
Valine	569 ± 79	565 ± 145	637 ± 91	577 ± 128	560 ± 59	510 ± 77	489 ± 45

Total IAA	1744 ± 209	1744 ± 471	2005 ± 268	1843 ± 380	1724 ± 175	1541 ± 233	1977 ± 308
Total DAA	3846 ± 397	3778 ± 941	4695 ± 549	4249 ± 814	4094 ± 404	3763 ± 512	4142 ± 403
Total AA	5589 ± 599	5522 ± 1407	6700 ± 806	6092 ± 1184	5818 ± 573	5304 ± 737	6119 ± 685

[1]All values are means ± SEM. n = 7 females and n = 6 males. Amino acid analysis by UPLC (Waters Corporation, Milford, MA). Values with different superscripts were significantly different, $P < 0.05$ determined by joint linear mixed effect model with sex and methionine intake as fixed effect terms and subject as a random effect term. Plasma AA concentrations were unaffected by sex ($P > 0.05$). IAA, indispensable amino acids; NIAA, non-indispensable amino acids.
[2]Methione and cysteine analyzed using UPLC-MS/MS whereby the MS/MS (Waters Xevo TQS mass spectrometer; Waters Corporation, Milford, MA) is coupled to UPLC (Waters H class UPLC; Waters Corporation, Milford, MA). The University of British Columbia.
